# Auditory Comprehension Deficits in Post-stroke Aphasia: Neurologic and Demographic Correlates of Outcome and Recovery

**DOI:** 10.3389/fneur.2021.680248

**Published:** 2021-08-12

**Authors:** Sandy J. Lwi, Timothy J. Herron, Brian C. Curran, Maria V. Ivanova, Krista Schendel, Nina F. Dronkers, Juliana V. Baldo

**Affiliations:** ^1^Veterans Affairs Northern California Health Care System, Martinez, CA, United States; ^2^Department of Psychology, University of California, Berkeley, Berkeley, CA, United States

**Keywords:** stroke, recovery, comprehension, temporal lobe, MRI, outcome

## Abstract

**Introduction:** One of the most challenging symptoms of aphasia is an impairment in auditory comprehension. The inability to understand others has a direct impact on a person's quality of life and ability to benefit from treatment. Despite its importance, limited research has examined the recovery pattern of auditory comprehension and instead has focused on aphasia recovery more generally. Thus, little is known about the time frame for auditory comprehension recovery following stroke, and whether specific neurologic and demographic variables contribute to recovery and outcome.

**Methods:** This study included 168 left hemisphere chronic stroke patients stroke patients with auditory comprehension impairments ranging from mild to severe. Univariate and multivariate lesion-symptom mapping (LSM) was used to identify brain regions associated with auditory comprehension outcomes on three different tasks: Single-word comprehension, yes/no sentence comprehension, and comprehension of sequential commands. Demographic variables (age, gender, and education) were also examined for their role in these outcomes. In a subset of patients who completed language testing at two or more time points, we also analyzed the trajectory of recovery in auditory comprehension using survival curve-based time compression.

**Results:** LSM analyses revealed that poor single-word auditory comprehension was associated with lesions involving the left mid- to posterior middle temporal gyrus, and portions of the angular and inferior-middle occipital gyri. Poor yes/no sentence comprehension was associated almost exclusively with the left mid-posterior middle temporal gyrus. Poor comprehension of sequential commands was associated with lesions in the left posterior middle temporal gyrus. There was a small region of convergence between the three comprehension tasks, in the very posterior portion of the left middle temporal gyrus. The recovery analysis revealed that auditory comprehension scores continued to improve beyond the first year post-stroke. Higher education was associated with better outcome on all auditory comprehension tasks. Age and gender were not associated with outcome or recovery slopes.

**Conclusions:** The current findings suggest a critical role for the posterior left middle temporal gyrus in the recovery of auditory comprehension following stroke, and that spontaneous recovery of auditory comprehension can continue well beyond the first year post-stroke.

Aphasia is a devastating language disorder that occurs in over 30% of patients who have a stroke (1, 2). Aphasia can impair an individual's ability to understand and produce language, leading to a number of social and functional challenges and decreased quality of life (3–5). Recovery from aphasia varies widely among individuals, and the factors predicting recovery are not well-understood. Current research on aphasia recovery has largely examined broad outcome measures such as overall aphasia severity, rather than specific aspects of speech and language (6–14). In particular, very little work has examined outcomes and recovery of auditory comprehension following stroke. This gap in knowledge is significant because comprehension deficits not only disrupt patients' functional abilities but can also compromise intervention and rehabilitation efforts. The present study examined the neurologic and demographic correlates of auditory comprehension outcome and recovery, using lesion-symptom mapping (LSM) and survival curve-based time compression.

Current knowledge on the recovery pattern of auditory comprehension following stroke is limited, as only a handful of studies have focused on this aspect of aphasia recovery. Moreover, when studies do focus on auditory comprehension, they typically only target the first several months post-stroke (note: in this paper, we use the term “recovery” to refer to spontaneous improvement over time and “outcome” to refer to a final chronic endpoint). Pickersgill and Lincoln (15) studied 56 chronic stroke patients (most of whom were an average of 3–4 months post-stroke) and found that most patients demonstrated significant recovery in auditory comprehension during the 2 months the study was conducted, with severely aphasic patients showing the greatest changes in auditory comprehension scores (15). Recovery after this 2-month assessment period was not examined. In another study, Mazzoni and colleagues assessed 45 stroke patients at 15 days, 1, 3–4, and once more at 6–7 months post-stroke. Findings indicated that spontaneous recovery of auditory comprehension occurred primarily within the first four months post-stroke (16). This finding is also supported by a small study with 22 mild-to-severe acute stroke patients who were given either the dictated commands test from the Boston Diagnostic Aphasia Exam or the sequential commands task of the Western Aphasia Battery at baseline and 90 days post-stroke. Results revealed that all of the patients had improved after 90 days, but no additional follow-ups were completed (17). In a more recent pilot study that assessed six patients at 2, 16, and 190 days post-stroke, results indicated that patients showed spontaneous recovery of auditory comprehension two-weeks post-stroke, but no significant changes were found at the 190-day/6-month assessment (18). Prins et al. (19) measured recovery of sentence comprehension and spontaneous speech up to a year post-stroke and found that patients continued to improve in comprehension (though not spontaneous speech) at each of the three assessments conducted over the year. In short, there is limited information on the trajectory of spontaneous recovery of auditory comprehension deficits associated with aphasia, particularly with respect to recovery after the first year, and the few results examining auditory comprehension recovery are mixed with respect to the degree and time course of recovery.

While many studies have attempted to identify the neural correlates of aphasia outcome and recovery, fewer studies have focused on the neural substrates of auditory comprehension specifically. In an early study by Selnes et al. (20), poor sentence comprehension outcome at 6 months post-stroke was associated with lesions in the left posterior superior temporal and supramarginal gyri. Similarly, Naeser et al. (21) found that sentence comprehension was most affected by posterior superior temporal gyrus lesions. This is supported by recent studies that similarly have found that the most critical regions for sentence comprehension were the left posterior superior temporal gyrus and inferior parietal cortex (22, 23). Dronkers et al. (24) found that comprehension of simple declarative sentences was dependent on the left posterior middle temporal gyrus; but a network of additional regions was critical for more complex syntactic processing, including left temporo-parietal cortex and lateral prefrontal regions. The importance of these regions for sentence comprehension are also supported in more recent lesion studies examining deficits in comprehension of complex sentences and syntactic structures (i.e., agrammatism). In these studies, patients with acute and chronic stroke similarly display more deficits in auditory sentencent studies that simila comprehension when damage was located in posterior middle temporal gyrus (25) and temporo-parietal cortex regions (26, 27), with one study also adding the importance of occipital and underlying white matter regions for completing auditory-visual sentence processing (28). With respect to single-word comprehension (29), Selnes et al. assessed patients at 1, 3, and 6 months post-stroke and found that even patients with damage to posterior superior temporal cortex showed good outcomes. Consistent with this finding, a recent study by Bonilha et al. (30) found rather that single-word comprehension deficits in chronic stroke patients were associated with lesions in the left inferior temporal and fusiform gyri and posterior temporal white matter. In sum, a number of different outcome studies have identified regions critical for single-word and sentence comprehension, but studies have typically focused on chronic outcome and either on single-word or sentence comprehension. Generally, sentence-level comprehension has been associated with left posterior temporo-parietal cortex and single-word comprehension with more inferior and sometimes anterior temporal lesions (31). In the current study, we had the opportunity to identify brain regions associated with both single-word auditory comprehension and two different types of sentence-level comprehension (sequential commands and yes/no questions) in a large group of patients, in order to determine the extent to which these different aspects of auditory comprehension are reliant on distinct brain regions.

With respect to demographic predictors, previous studies that have assessed general aphasia recovery have found that variables such as younger age, being female, higher educational attainment, and lower initial stroke severity are associated with better recovery (5, 32–37). However, the role of these demographic factors in the recovery and outcome of auditory comprehension deficits in aphasia more specifically has not been well-studied and findings have been mixed. Pickersgill and Lincoln (15) reported small or no correlations between age and recovery of auditory comprehension in chronic aphasia patients, while Lazar et al. (17) found no age, education, or gender associations with outcome or recovery of auditory comprehension at baseline or 3 months post-stroke. In contrast, a larger study with 173 acute stroke patients tested on a battery of language tests, including a word-recognition auditory comprehension task, found that acute stroke patients with at least 12 years of education had higher auditory comprehension outcome scores than those with less than a high school education (34).

The present study addressed some of the gaps in the current literature on outcome and recovery of auditory comprehension by analyzing retrospective data from a large, well-characterized group of stroke patients with a wide range of auditory comprehension deficits. First, we utilized univariate and multivariate LSM, a well-established lesion mapping approach (38), to examine neuroanatomical associations of three different aspects of auditory comprehension, including both single-word and sentence-level comprehension. Second, in a subgroup of patients who were tested at multiple timepoints, we quantified the trajectory of auditory comprehension recovery using survival curve-based time compression. This analysis allowed us to capture patients' longitudinal trajectory of recovery during and beyond the first year post-stroke. Last, we analyzed the impact of demographic variables on outcome and recovery of auditory comprehension deficits following stroke.

## Methods

### Participants

#### Chronic Outcome Group

Language and brain imaging data were retrospectively analyzed from 168 patients in our stroke research database who met the following criteria: History of a single left hemisphere stroke; pre-morbidly right-handed; native English speaker; at least 12 months post-stroke; minimum eighth grade education, no other neurologic history; no severe psychiatric history (e.g., bipolar disorder, schizophrenia); concurrent brain imaging; no visual agnosia; normal/corrected-to-normal vision and hearing, and ability to comply with task instructions. The primary reasons for exclusion from this retrospective analysis were: (1) the presence of right hemisphere and/or multiple strokes, (2) no brain imaging data, and (3) no auditory comprehension subtest score. Patients with small right hemisphere lacunae were not excluded.

Based on the Western Aphasia Battery [WAB; (39)], the sample included 45 patients with Broca's aphasia, 14 patients with Wernicke's aphasia, 6 patients with conduction aphasia, 47 patients with anomic aphasia, 4 patients with global aphasia, 3 patients with transocrtical sensory aphasia, 1 patient with transcortical motor aphasia, and 48 patients who scored within-normal limits (WNL; see Table 1 for patient characteristics). WNL status is based on a cut-off score of 93.7/100 on the WAB. This cut-off score was taken from the WAB manual, which derived these scores by comparing stroke patients to a control sample. These WNL patients had a prior history of clinical aphasia and residual deficits, but their symptoms were too mild to be detected by the WAB.

**Table 1:** Participant characterization for chronic outcome and recovery groups.

	**Chronic outcome group**	**Recovery group**	**Statistical test**
*N*	168	44	
**Age**			
Mean (SD)	61.2 (11.2)	62.2 (10.2)	*t*(210) = 0.56, *p* = 0.574
Range	31–86	41–80	
**Education**			
Mean (SD)	14.9 (2.4)	13.9 (2.2)	*t*(210) = −2.39, *p* = 0.018
Range	12–22	9–20	
**Gender (% male)**	79%	80%	*X^2^*(1, 210) = 0.66, *p* = 0.418
**Months post-stroke**			
At initial test	–		
Mean (SD)	–	26.7 (42.1)	
Range	–	1–219	
At final test			
Mean (SD)	51.4 (54.0)	68.8 (72.5)	*t*(210) = −1.71, *p* = 0.089
Range	12–27	5–328	

*Only the recovery group completed multiple WAB tests, and thus no initial scores are reported for the chronic outcome group*.

#### Recovery Group

For the recovery analysis, we analyzed data from a group of 44 patients who were tested on the WAB on at least two different timepoints, separated by at least 1 month without any intervening treatment (see Table 1 for patient characteristics). Because this was a retrospective analysis, patients were not systematically tested during specific acute and chronic timepoints, but rather, this analysis made use of existing data from patients who were tested more than once at various points in their recovery. In this way, we were able to measure recovery curves across many months and years post-stroke. Patients who reported any new or additional neurologic events between behavioral test sessions were not included in this study. To avoid ceiling effects, we only included patients with auditory comprehension subscores <9 out of a possible 10 (as derived from the WAB manual) in their initial test session. The average time interval between patients' first and last WAB test session was 42.2 months (*SD* = 58.4; range 2–284). The average number of test sessions administered in this subgroup was 2.7 (SD = 1.0; range 2–6). Based on their final outcome scores, this subset included 9 patients with an anomic aphasia, 14 patients with Broca's aphasia, 6 patients with conduction aphasia, 1 patient with global aphasia, 2 patients with transcortical sensory aphasia, 10 patients with Wernicke's aphasia, and 2 WNL patients. For more details on the first and final WAB scores received by the recovery group, see Table 2.

**Table 2:** Participant WAB subtest scores (initial and final) for chronic outcome and recovery groups.

	**Yes/no questions**		**Single-word recognition**		**Sequential commands**		**Aphasia quotient**		**Overall comprehension score**	
	**Initial**	**Final**	**Initial**	**Final**	**Initial**	**Final**	**Initial**	**Final**	**Initial**	**Final**
**Chronic outcome**										
Mean (SD)	–	54.9 (9.1)	–	50.9 (13.3)	–	58.3 (22.7)	–	70.6 (28.3)	–	8.2 (2.1)
Range	–	0–60	–	8–60	–	0–80	–	9.1–100	–	0.9–10
**Recovery**										
Mean (SD)	48.0 (11.3)	50.2 (10.7)	39.4 (15.2)	44.5 (14.4)	35.1 (22.4)	46.3 (72.8)	50.5 (25.3)	55.8 (25.5)	6.1 (2.1)	7.0 (2.0)
Range	15–60	12–60	4–60	9–60	0–76	4–80	6.3–92.6	10.7–97.9	1.8–8.8	1.8–10.0
p-value		*P* = 0.004		*P* = 0.005		*P* = 0.001		*P* = 0.002		*P* < 0.001

*The recovery group (n = 44) completed multiple WABs while the chronic group (n = 168) only completed one. Thus, for the chronic group initial tests were also their final test. Independent t-tests were conducted to examine group differences between final test scores. Recovery group final scores were on average, obtained 69 months post stroke, while the chronic stroke group final scores were on average, obtained at 51 months post stroke*.

Informed consent was obtained from all patients in the study. The study was carried out in accordance with the Helsinki Declaration and approved by the VA Northern California Institutional Review Board.

## Materials and Procedures

### Auditory Comprehension Testing

Patients were administered the WAB by a licensed speech-language pathologist or trained neuropsychologist as part of a larger neuropsychological battery for research purposes. The WAB is a speech and language battery that includes separate subtests that measure speech fluency, naming, repetition, and comprehension. Only patients who could comply with test instructions were included in these analyses. The present study analyzed data from the three WAB auditory comprehension subtests: (1) yes/no questions −20 items (e.g., “Is this a hospital?”), (2) single-word recognition −60 items (e.g., point to the cup), and (3) sequential commands −11 items (e.g., point to the book and the comb).

### Brain Imaging and Lesion Reconstructions

Patients underwent brain imaging at least 3 months post-stroke, when lesion site and size are stabilized. Patients' lesions were reconstructed from 3D MRI T1 scans, or 3D CT scans when MRI was contraindicated. Imaging was acquired close to the time of the first WAB test session. For 59 patients, high-resolution T1-weighted 3D MRI scans were obtained on a 1.5 T Phillips Eclipse scanner. T1-weighted images were acquired with a Spoiled Gradient Recall (SPGR) coronal sequence (TR/TE/FA = 15 ms /4.47 ms / 35°, FOV = 256 × 240 × 256, 0.94 × 1.3 × 0.94 mm^3^ voxels). For 14 patients, anatomical scans were obtained on a 3T Siemens Verio scanner (Syngo MR B17) with a 12-channel phased-array head coil. One high-resolution 3T T1 MPRage anatomical image was acquired for each subject (TR/TE/FA = 2,200 ms/1.62 ms/9°, FOV = 256 × 192 × 256 mm, 1 × 1 × 1 mm^3^ voxels, inversion = 900 ms, bandwidth = 343 Hz/voxel, and GRAPPA factor = 2) along with a T2 image (TR/TE/FA = 3,000 ms/409 ms/120°, FOV = 256 × 192 × 256 mm, 1 × 1 × 1 mm^3^ voxels, bandwidth = 751 Hz/voxel) and a FLAIR image (TR/TE/FA = 6,000 ms/388 ms/120°, FOV = 250 × 250 × 192 mm, 0.488 × 0.488 × 1 mm^3^ voxels, inversion = 2,100 ms, bandwidth = 781 Hz/voxel). Patient lesions were outlined directly on the patient's T1 digital MRI image using MRIcron (40) and then registered with the MNI template using the standard nonlinear spatial normalization procedure from SPM5 (Statistical Parametric Mapping, Wellcome Trust Centre for Neuroimaging). A cost function masking procedure was used to avoid distortions due to the presence of the lesion (41). T2 and FLAIR images were yoked to the T1 images in MRIcron to verify the extent of the lesion.

When 3D digital MRI images were not available, lesions were drawn from hard-copy CT (*n* = 7) or MRI films (*n* = 88). The CT scanner was a Siemens Somatom Emotion 16 CT scanner with 3 × 3 × 3 mm^3^ imaging, and MRI images were collected on the 1.5 T scanner described above. Lesions were outlined onto an 11-slice, standardized template (based on the atlas by DeArmond et al. (42) by a board-certified neurologist who was blind to the patient's clinical presentation as well as the predictions of the study. Reliability with this technique has been previously demonstrated (43, 44). The brain templates were then digitized and non-linearly transformed into MNI space (45) with SPM5. This transformation was achieved using 50 control point pairs to match anatomical features on the two templates. Slices were then aligned using a local weighted mean transformation implemented using the *cpselect, cp2tform*, and *imtransform* Matlab imaging toolbox functions.

**Figure 1:**
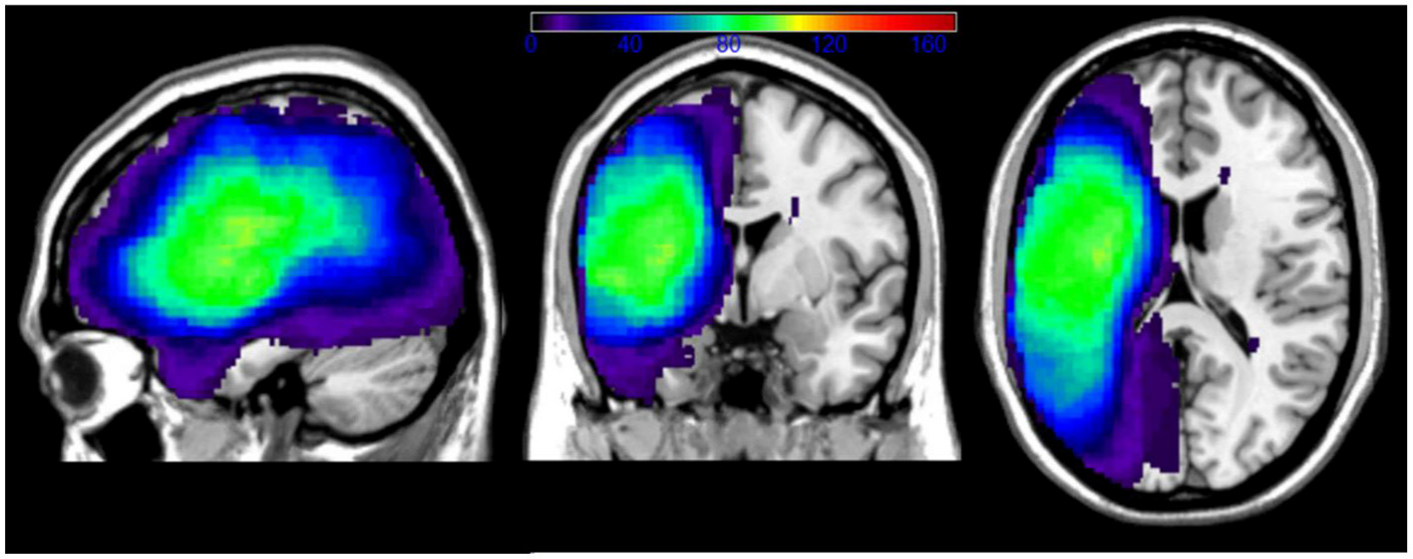
Overlay of patients' lesions in the chronic outcome LSM analyses. Color bar indicates number of patients with lesion overlap. Patients with right hemisphere lacunae were not excluded from our sample.

An overlay map of patients' lesions is shown in Figure 1. As illustrated, the extent of coverage in the analyses included much of the left cerebral hemisphere and underlying white matter, predominantly in the middle cerebral artery distribution. The average lesion volume for the sample was 121.8cc.

### Data Analysis

#### Lesion-Symptom Mapping

To identify the gray and white matter correlates of auditory comprehension outcome scores, patients' behavioral data were related to their lesion reconstructions using univariate and multivariate lesion-symptom mapping (38) (software freely available at https://www.nitrc.org/projects/clsm/). With univariate LSM, linear regression functions are estimated at every voxel, comparing behavioral performance (here, auditory comprehension scores) in individuals with and without a lesion in each voxel. Analyses were limited to those voxels that had at least five individuals with a lesion in that voxel in order to minimize biased regression parameter estimates. Maximum-value permutation testing was used to determine the critical *t*-value threshold, based on 10,000 iterations and alpha set at 0.05 (46). This is a relatively conservative method to account for the large number of statistical comparisons across the brain. We also re-ran the analyses using multivariate LSM, using support vector regression (SVR) with the same parameters above, namely 10,000 iterations with alpha set at 0.05 to determine the critical *t*-value threshold (47, 48). SVR has high spatial accuracy and a spatially compact solution (38). All analyses included age, gender, months post-stroke, overall aphasia severity (WAB overall score), and lesion volume as covariates. Identification of the brain regions associated with the significant voxels in each map was made with the AAL atlas template in MRIcron.

#### Longitudinal Recovery Slopes

Using auditory comprehension data from multiple time points, each patient's recovery was computed as a single longitudinal slope using mixed-effects linear models (R, “lme4” package). Individual intercepts were used to search for the best transformation of auditory comprehension recovery time (of 10 different families). Best fit to the longitudinal data is reported, based on the Bayesian Information Criterion (BIC) model score. Slopes computed from the time transformation with the lowest BIC score were then used in the LSM and behavioral analyses of recovery.

#### Demographic Analysis

Associations between patient demographic variables and auditory comprehension outcome scores were examined with linear regressions, with outcome scores as the dependent variable and months post-stroke, age, gender, and education as independent variables. Associations between patient demographic variables and auditory comprehension recovery slopes were examined with linear mixed effects models, with longitudinal recovery slopes as the dependent variable and months post-stroke, age, gender, and education as fixed effects. Patients were entered as random effects.

## Results

### LSM Analysis of Auditory Comprehension

**Figure 2:**
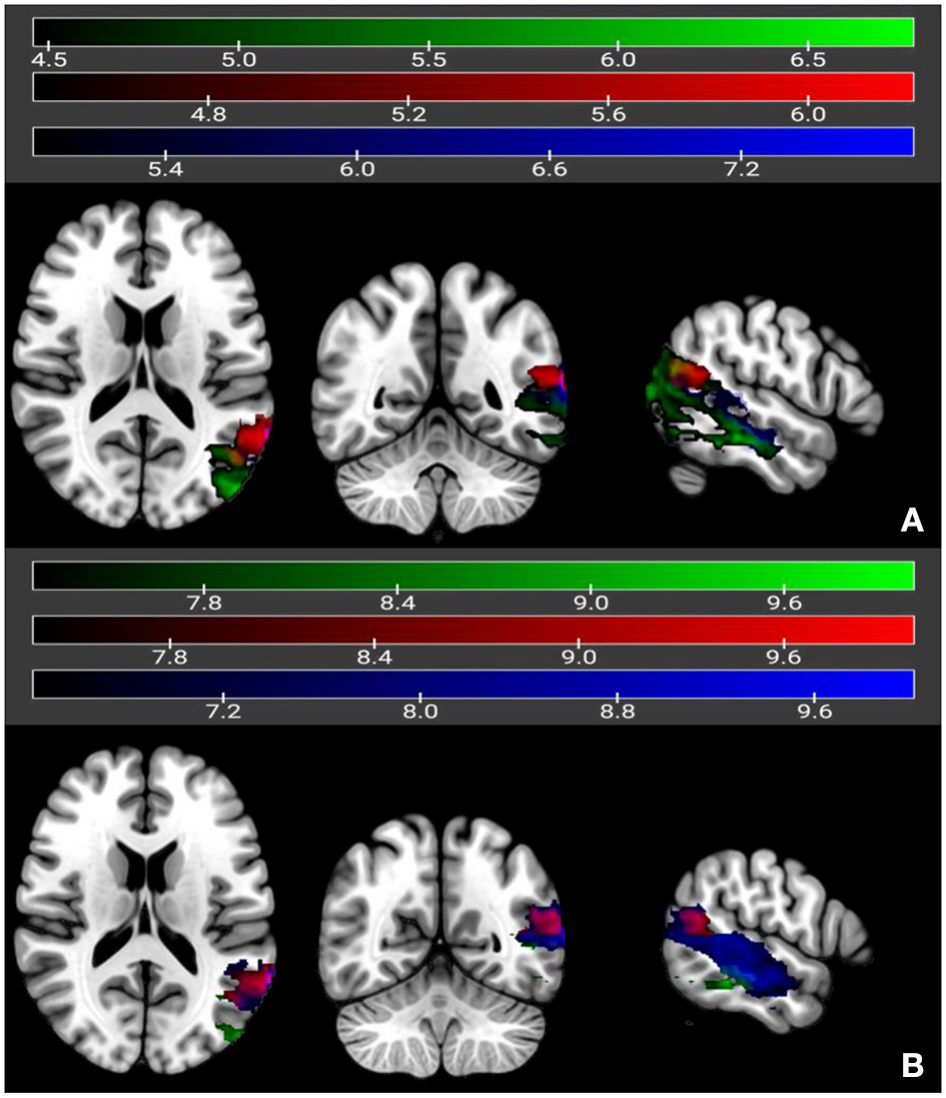
Univariate **(A)** and multivariate **(B)** LSM maps showing neural correlates of yes/no questions (in dark blue), single-word comprehension (in green), and sequential commands (in red). Regions overlapping between single-word comprehension and yes/no questions are in light blue, and regions overlapping between single-word comprehension and sequential commands are in magenta. Regions overlapping across all three subtests are in yellow. Color bars reflect *t*-values.

The univariate LSM analysis identified distinct brain regions that were critically associated with the chronic outcome scores of the different auditory comprehension tasks, where the critical *t-*threshold was surpassed at *p* < 0.05. As seen in Figure 2, poor outcome for single-word auditory comprehension was associated with lesions in the left mid- to posterior middle and inferior temporal gyri (*t*-max = 6.97 at −60, −38, −14, cluster size = 4,583, *t*-threshold = 4.46) as well as small portions of the left angular and inferior-middle occipital gyri. For comprehension of yes/no questions, poor outcome was associated with lesions involving the left mid- to posterior middle temporal gyrus (*t*-max = 7.73 at −66, −22, −16, cluster size = 3,377, *t*-threshold = 4.99), and included portions of the left posterior superior temporal gyrus and posterior inferior temporal gyrus. Poor outcome scores for comprehension of sequential commands was associated with lesions in the left posterior middle temporal gyrus (*t*-max = 6.21 at −64, −54, 14, cluster size =854, *t*-threshold = 4.45), with some involvement of the left superior temporal gyrus and angular gyrus. There was a small region of common overlap across these three auditory comprehension subtests, located in the left posterior middle temporal gyrus.

Multivariate LSM analysis using SVR identified similar regions to the univariate LSM results, again where the critical *t-*threshold was surpassed at *p* < 0.05, for yes/no comprehension (*t*-max = 10.0 at −66, −22, −14, cluster size = 4,913, *t*-threshold = 6.43) and sequential commands (*t*-max = 10.0 at −64, −54, 14, cluster size = 770, *t*-threshold = 7.40). Single word auditory comprehension still fell in the posterior middle temporal gyrus, although the focus was more posterior than that identified with univariate LSM. The cluster size was also smaller (*t*-max = 10.0 at −40, −76, 20, cluster size = 1,866, *t*-threshold = 7.27). The region of common overlap across the auditory comprehension subtests was in the left posterior middle temporal gyrus as seen in the univariate LSM analyses.

Additional LSM analyses were also conducted on each of the three comprehension outcome scores, with the two other comprehension tasks included as covariates (e.g., examining single-word comprehension with scores from yes/no questions and sequential commands added as covariates), but there was not enough variability to generate any significant voxels.

Neither univariate nor multivariate LSM identified any significant voxels associated with the recovery slopes, likely due to the small sample size in the recovery group. Univariate LSM typically requires a minimum of 50 participants to generate significant, reliable findings (depending on effect size), and multivariate LSM typically requires 60–70 participants (38).

### Analysis of Recovery Slopes

**Figure 3:**
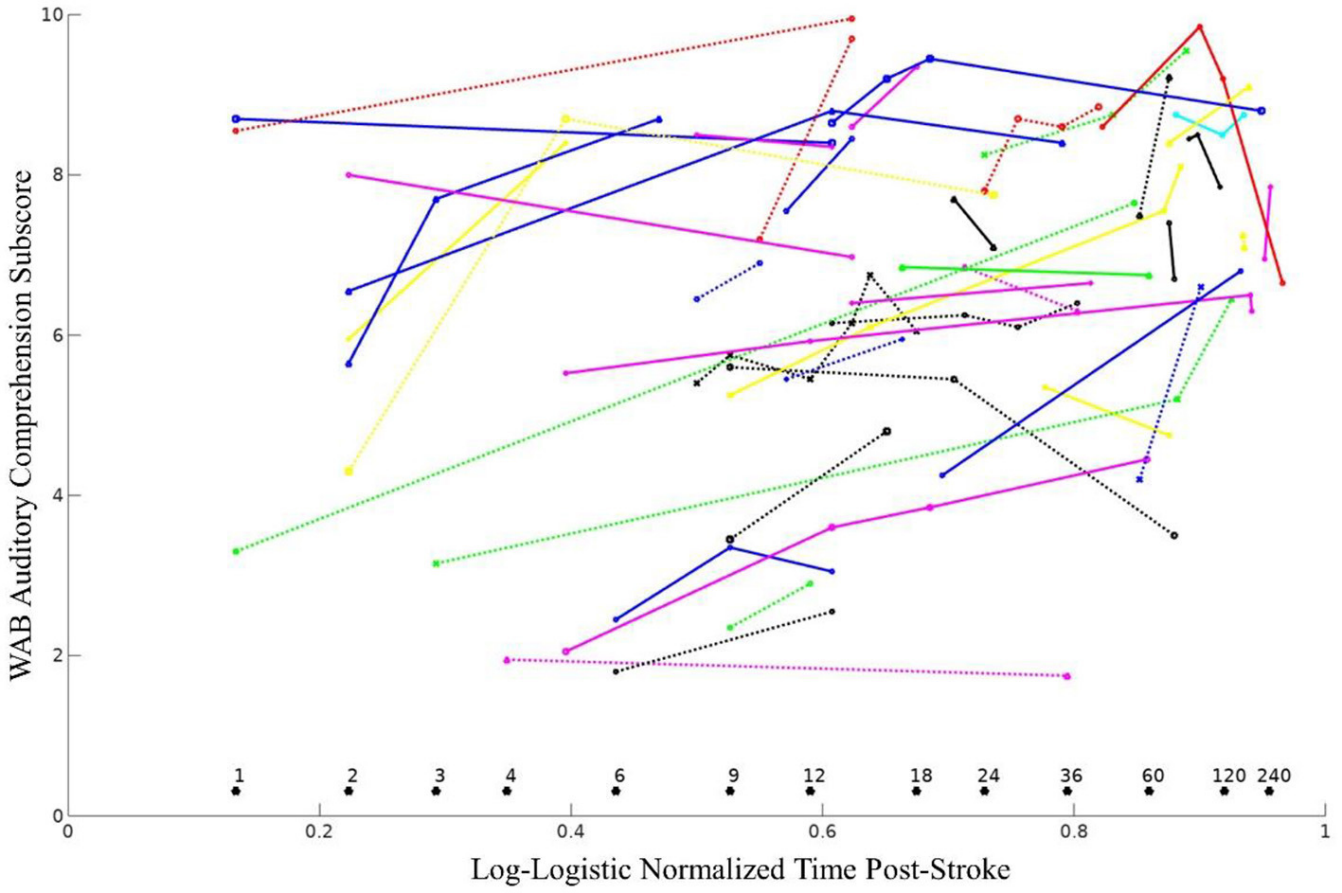
WAB auditory comprehension subscores over time shown as distinct lines for all 44 individuals. Months post-stroke are reported above each asterisk (*). The log-logistic time compression provided an optimal transformation for examining recovery of auditory comprehension, using the following equation, where MPO represents months post-onset: $f\left(MPO\right)=\frac{1}{1+{\left(\frac{MPO}{8}\right)}^{-0.9}}$.

Longitudinal recovery of overall auditory comprehension (collapsed across all three subtests) was examined using survival curve-based time compression with ten different time compressions (see Table 3). Exponential log, weibull, lomax, and log-logistic classes produced the models with the best fit (BIC = 464.4–464.8). The overall estimated best slope for the log-logistic model was 3.71 (SD = 0.60) The log-logistic normalized time suggests that approximately 60% of improvement in auditory comprehension occurred by 12 months post-stroke, and approximately 80% of improvement occurred by 36 months post-stroke (see Figure 3). There was approximately the same amount of improvement (~7%) in auditory comprehension between 18 and 24 months post-stroke as there was between 60 and 120 months post-stroke.

**Table 3:** Survival curve-based analysis fit (BIC) for different time compression models.

	**BIC**
Exponential log	464.4
Weibull	464.6
Lomax	464.8
Log Logistic	464.8
Gamma	466.4
Exponent	466.3
Gompertz	466.3
LogPower	465.4
InvGamma	465.1
LogCauchy	465.0

*Lower values indicate better fit*.

We also attempted the same survival curve-based analysis using separate recovery slopes for the three individual auditory comprehension subtest scores (yes/no questions, single-word comprehension, and sequential commands), but the optimal models had poor model fit (i.e., unclear group recovery slopes), likely due to increased noise in these subtests relative to the overall comprehension score. This in turn reduced the models' ability to effectively transform the data and accurately detect longitudinal slopes.

### Impact of Demographic Variables on Outcome and Recovery

First, we analyzed the relationship between demographic variables (age, gender, education, and months post-onset) and chronic outcome scores for the three different comprehension subtests. Regression analyses revealed that higher education was positively associated with higher yes/no question scores [*B* = 0.61, *SE* = 0.29, β = 0.16, *p* = 0.041, 95% CI = (0.02–1.18)], single-word recognition scores [*B* = 1.26, *SE* = 0.42, β = 0.22, *p* = 0.003, 95% CI = (0.43–2.10)], sequential commands [*B* = 2.57, *SE* = 0.72, β = 0.27, *p* < 0.001, 95% CI = (1.15–3.99)], and the overall auditory comprehension score [*B* = 0.22, *SE* = 0.07, β = 0.25, *p* < 0.001, 95% CI = (0.09–0.35)]. While age trended toward significance across all measures (*p* < 0.14), none of the other demographic variables were associated with any of the comprehension subtest outcome scores.

Secondly, we assessed the relationship between demographic variables and patients' recovery curves. Longitudinal recovery curves were significantly associated with time post-stroke [*B* = 0.007, *SE* = 0.003, β = 2.43, *p* = 0.02, 95% CI = (0.41–0.80)], as greater chronicity was associated with better recovery. Gender [β = −1.41, *SE* = 0.74, *t*(40) = −1.90, *p* = 0.06, 95% CI = (−2.98, 0.03)], and age [β = −0.04, *SE* = 0.02, *t*(42) = −1.78, *p* = 0.08, 95% CI = (−0.09, −0.01)] trended toward significance, with male gender being associated with better recovery and higher age associated with poorer recovery. In contrast to the outcome scores, education showed no association with longitudinal recovery [β = −0.12, *SE* = 0.13, *t*(40) = −0.93, *p* = 0.36, 95% CI = (−0.42, 0.10)].

## Discussion

Auditory comprehension is often negatively impacted in stroke-related aphasia, but prior recovery studies of aphasia have typically focused on broad measures, such as overall aphasia severity. Also, very few studies have investigated recovery beyond the first year post-stroke (35). In the current study, we specifically examined recovery and outcome of auditory comprehension over an extended time period, including the impact of lesion site and demographic variables. First, we used univariate and multivariate lesion-symptom mapping (LSM) to identify the neural correlates of outcome for three different aspects of auditory comprehension in a large sample of 168 stroke patients (38). Lesion correlates for all three tasks overlapped in a small portion of left posterior middle temporal cortex, but there was substantial divergence across the tasks as well: Single-word auditory comprehension was uniquely associated with inferior temporal cortex as well as inferior middle-occipital regions; answering yes/no questions was associated primarily with mid- to posterior middle temporal gyrus; and comprehending sequential commands was associated with the left posterior middle temporal gyrus, with some involvement of left superior temporal and angular gyri. These findings provide context for discrepant results in the literature on auditory comprehension that may arise from the use of different types of tasks. Consistent with previous findings (30), tasks involving picture/object stimuli were associated with more inferior temporal cortical regions, while sentence-level tasks (which likely engage verbal working memory) tapped into more superior temporal and inferior parietal cortices.

The only brain region common to all three auditory comprehension tasks was a small region of posterior middle temporal gyrus, a highly-interconnected region that we and others have shown to be a critical hub for lexical-semantics (25, 49–52). While some studies have suggested that single-word comprehension is not dependent on posterior temporal cortex (29), our findings are consistent with recent studies by Fridriksson et al. and Kristinsson et al. (25, 53). Fridriksson and colleagues reported that both single-word and sentence comprehension were significantly impacted by lesions in the posterior superior and middle temporal gyri in chronic stroke patients, while Kristinsson and colleagues found that damage to the posterior middle temporal gyrus was the strongest predictor of overall auditory sentence comprehension performance in acute stroke patients. Studies in acute stroke patients have also implicated the posterior superior temporal gyrus in single-word comprehension, though not the posterior middle temporal gyrus as we did (54). Other studies, particularly with cases of progressive aphasia, have suggested that more anterior temporal regions are critical for single-word comprehension (31, 55). We were not able to directly address the role that the anterior temporal lobe plays in auditory comprehension due to the predominance of middle cerebral artery strokes in our patient sample.

Our study is one of the first to examine the longitudinal recovery of auditory comprehension specifically, and our results revealed that recovery occurs over a longer period than typically thought or previously investigated. Our findings revealed that, counter to common clinical wisdom, spontaneous recovery of auditory comprehension (i.e., without intervention) can continue well-beyond the first year post-stroke. Prior studies of auditory comprehension recovery have largely examined recovery over the span of only a few months post-stroke (16, 18, 19), while even studies examining language recovery more broadly only followed chronic stroke patients up to a year post-stroke. We also found that chronicity was predictive of the slope of recovery of auditory comprehension, but other factors such as age, gender, and education were not predictive (or trend only). By expanding the time window to include many years post-stroke, our study was able to demonstrate a longer course of recovery. Understanding the time course of auditory comprehension recovery has implications for treatment, as it supports the idea that patients can still show spontaneous improvement beyond the first year of recovery and indirectly supports the growing use of speech-language interventions during the chronic phase of stroke (56, 57).

Notably, recovery in our sample was not due to any intervening speech and language treatment, as this was a part of our exclusion criteria. Patients' spontaneous recovery over time thus likely reflected (at least in part) natural regeneration and the recruitment and activation of undamaged or compensatory neural regions (58, 59). Regarding the mechanisms that may undergird recovery of auditory comprehension specifically, few studies have examined non-speech related treatments that may contribute to improvement. The literature that is available in this area suggests that aspects such as exercise (60–62), access to quality medical care and fewer comorbidities (62, 63), managing psychiatric sequelae (64, 65) and social support (66–68) can all impact stroke patients' overall cognition and functioning. Thus, it is possible that such factors contributed to the spontaneous improvement of auditory comprehension in this sample, but additional work is necessary to explore this possibility.

Although there was evidence of recovery in auditory comprehension beyond the first year post-stroke, our data also showed that some elderly patients many years post-stroke sometimes exhibited declines in auditory comprehension. This diminution could be due to medical factors such as progressive cognitive decline that are more common in this aging population (69, 70). Future studies should devote effort to understanding these longer-term recovery patterns and the individual factors that can lead to continued or suspended long-term recovery from aphasia. Our use of longitudinal recovery slopes provides a way to examine auditory comprehension in a large sample of patients at various points in their stroke recovery. Our analyses revealed that the exponential log, weibull, lomax, and log-logistic time compressions best captured recovery over time, with log-logistic compression providing the most stable and interpretable results. This method also reveals additional avenues for assessing variables associated with aphasia recovery. By providing an approach for examining retrospective data, we hope this work will facilitate future collaborations and lead to larger aggregate datasets that can readily capture the neuroanatomical regions that are most critical for auditory comprehension recovery.

With respect to outcome, education was strongly associated with higher outcome scores on each of the three auditory comprehension tasks. This finding builds upon a prior study that found that acute stroke patients (within 24 h of stroke onset) with at least 12 years of education had higher scores on an auditory comprehension word-recognition task (34). However, studies have also found no associations between education and auditory comprehension outcome/recovery (15, 17), possibly due to a lack of statistical power. Identifying demographic factors associated with outcome and recovery is an important area of future research, as understanding which people may be more vulnerable to poor auditory comprehension recovery can help provide more accurate prognoses and lead to earlier intervention.

Strengths of the current study include our examination of both neuroanatomical and demographic factors associated with outcome scores of auditory comprehension in a large sample of well-characterized patients. We identified brain regions associated with auditory comprehension using both univariate and multivariate LSM. Recent empirical studies have suggested that the tandem use of both types of LSM is optimal for confirming critical foci (38). Both methods were consistent with respect to the critical foci identified in left mid-posterior temporal cortex, with the only exception being a somewhat more posterior middle temporal focus identified with multivariate LSM for single-word comprehension. Another strength of our study includes our analysis of recovery slopes many months and years post-stroke, which allowed us to demonstrate that spontaneous recovery continues beyond the first year.

One limitation in the current study was the smaller sample size of our recovery subgroup, which was not sufficiently large enough to utilize LSM to identify brain regions significantly associated with patients' recovery slopes (38). Also, because this was a retrospective analysis, patients were not consistently tested at specific timepoints during acute and chronic phases of stroke. Such evidence has not been forthcoming in previous longitudinal studies due to the difficulty and time required to follow a sufficiently large group of stroke patients over many years. Current efforts are underway at our site and others to amass large-scale stroke samples that include patients tested in both acute and chronic phases of stroke (71). Another consideration is that we cannot rule out the possibility that practice effects played a role in increasing scores on the WAB over time. However, practice effects in language testing are minimal, including the WAB (35, 36). In terms of demographics, the recovery group differed from the outcome group in their level of education, so it is possible that with a larger sample, education may be found to play a larger role in the recovery of auditory comprehension as well. The current study also did not address variables associated with language recovery in response to specific speech-language interventions, as we focused here on spontaneous recovery and outcome [for a review of intervention-based recovery, see (72)]. Another limitation is that we did not have tractography or functional imaging data available to address, for example, the role of the right hemisphere in supporting recovery of auditory comprehension (73, 74). Saur et al. (13) studied auditory comprehension recovery with fMRI in a group of 14 aphasic patients and found evidence of distinct patterns of activation associated with recovery during different phases of recovery. Last, LSM analyses of individual auditory comprehension subtests did not detect significant voxels when we covaried for the other comprehension subtests (e.g., examining single-word auditory comprehension with yes/no questions and sequential command subtests included as covariates). The high level of spatial overlap between the three tests made it difficult for the permutation tests to set an effective threshold, a statistical challenge made more difficult by adding the other two highly correlated tests as covariates. Nonetheless, the primary LSM analyses did show spatial divergence across tasks, as well as regions of common overlap.

## Conclusions

This study was one of the first to examine neuroanatomic and demographic variables associated with recovery and outcome of auditory comprehension after stroke. LSM analyses revealed that different aspects of auditory comprehension, including both single-word and sentence-level comprehension, were associated with distinct portions of left mid-posterior temporal cortex, with a common region of overlap in the left posterior middle temporal gyrus. These findings provide context for discrepant results in the literature on auditory comprehension that may arise from the use of different types of tasks. We found that tasks involving picture/object stimuli were associated with more inferior temporal cortical regions, while sentence-level tasks (which likely engage verbal working memory) tap more superior temporal and inferior parietal cortices. Higher education was also found to be associated with higher auditory comprehension outcome scores, while age and gender were not. Our findings also revealed that spontaneous recovery of auditory comprehension continues beyond the first year post-stroke.

## Data Availability Statement

The datasets presented in this article are not readily available because these data were collected at a VA (Veterans Affairs) facility, which does not readily allow data-sharing. Requests to access the datasets should be directed to juliana.baldo@va.gov.

## Ethics Statement

The studies involving human participants were reviewed and approved by VA Northern California Health Care System Institutional Review Board. All participants provided their written informed consent to participate in this study.

## Author Note

JB, Veterans Affairs Northern California Health Care System, Martinez, CA. SL, Veterans Affairs Northern California Health Care System, Martinez, CA. TH, Veterans Affairs Northern California Health Care System, Martinez, CA. BC, Veterans Affairs Northern California Health Care System, Martinez, CA. MI, University of California, Berkeley. KS, Veterans Affairs Northern California Health Care System, Martinez, CA. ND, University of California, Berkeley.

## Author Contributions

JB, ND, and MI contributed to conception and design of the study. TH, BC, KS, and SL organized the database. SL and TH performed the statistical analyses. SL wrote the first draft of the manuscript. JB, KS, and TH wrote sections of the manuscript. All authors contributed to the article and approved the submitted version.

## Conflict of Interest

The authors declare that the research was conducted in the absence of any commercial or financial relationships that could be construed as a potential conflict of interest.

## Publisher's Note

All claims expressed in this article are solely those of the authors and do not necessarily represent those of their affiliated organizations, or those of the publisher, the editors and the reviewers. Any product that may be evaluated in this article, or claim that may be made by its manufacturer, is not guaranteed or endorsed by the publisher.
